# Exome sequencing of a colorectal cancer family reveals shared mutation pattern and predisposition circuitry along tumor pathways

**DOI:** 10.3389/fgene.2015.00288

**Published:** 2015-09-15

**Authors:** Suleiman H. Suleiman, Mahmoud E. Koko, Wafaa H. Nasir, Ommnyiah Elfateh, Ubai K. Elgizouli, Mohammed O. E. Abdallah, Khalid O. Alfarouk, Ayman Hussain, Shima Faisal, Fathelrahamn M. A. Ibrahim, Maurizio Romano, Ali Sultan, Lawrence Banks, Melanie Newport, Francesco Baralle, Ahmed M. Elhassan, Hiba S. Mohamed, Muntaser E. Ibrahim

**Affiliations:** ^1^Faculty of Medicine, University of KhartoumKhartoum, Sudan; ^2^Department of Molecular Biology, Institute of Endemic Diseases, University of KhartoumKhartoum, Sudan; ^3^International Centre for Genetic Engineering and BiotechnologyTrieste, Italy; ^4^Weill Cornell Medical CollegeDoha, Qatar; ^5^University of SussexBrighton, UK

**Keywords:** colorectal cancer, *ELAVL1/HuR*, NFkB, exome sequencing, network analysis, pathway analysis

## Abstract

The molecular basis of cancer and cancer multiple phenotypes are not yet fully understood. Next Generation Sequencing promises new insight into the role of genetic interactions in shaping the complexity of cancer. Aiming to outline the differences in mutation patterns between familial colorectal cancer cases and controls we analyzed whole exomes of cancer tissues and control samples from an extended colorectal cancer pedigree, providing one of the first data sets of exome sequencing of cancer in an African population against a background of large effective size typically with excess of variants. Tumors showed *hMSH2* loss of function SNV consistent with Lynch syndrome. Sets of genes harboring insertions–deletions in tumor tissues revealed, however, significant GO enrichment, a feature that was not seen in control samples, suggesting that ordered insertions–deletions are central to tumorigenesis in this type of cancer. Network analysis identified multiple hub genes of centrality. *ELAVL1/HuR* showed remarkable centrality, interacting specially with genes harboring non-synonymous SNVs thus reinforcing the proposition of targeted mutagenesis in cancer pathways. A likely explanation to such mutation pattern is DNA/RNA editing, suggested here by nucleotide transition-to-transversion ratio that significantly departed from expected values (*p*-value 5e-6). *NFKB1* also showed significant centrality along with *ELAVL1*, raising the suspicion of viral etiology given the known interaction between oncogenic viruses and these proteins.

## Background

Colorectal carcinoma (CRC) is a universally prevalent cancer and a leading cause of death in the world. The overwhelming heterogeneity of cancer has always been the focus of many genetic studies. This complexity is notable at macroscopic, microscopic, and molecular levels. Colorectal cancer can be sporadic, or may show familial predisposition that is due to identifiable single gene mutations with Mendelian or near Mendelian segregation such as Hereditary Non-Polyposis Colorectal Cancer and Familial Adenomatous Polyposis. A remaining percentage of colorectal cancers are not familial but have an inherited tendency (Hendon and DiPalma, 2005; Bodmer, 2006; Fearson, 2011). It has been proposed that much of this inherited tendency may be due to the cumulative effects of low frequency, low penetrance variants, and possibly explained by the “rare variant hypothesis” (Kemp et al., 2004; Bodmer, 2006). The field of oncology is dominated by two competing theories that strife to explain the complex phenomenon of cancer: the somatic mutation theory and tissue organization field theory (Rosenfeld, 2013). Both theories appreciate the complex etiology and multiple molecular contributors to cancer phenotypes, but hitherto lack obvious translational impact and a lucid understanding of triggers of cancer. Furthermore, carcinogenesis is still viewed as a random sequel of changes that attains levels of organization through selective fitness of the tumor, although this scenario may raise other questions to account for situations where tumors show pronounced pathology at early stages or mutations targeting certain critical biological pathways.

Genetic and epigenetic changes in colorectal cancer encompass a wide range of alterations. The Cancer Genome Atlas Network (2012) conducted a genome-scale analysis of 276 samples to characterize somatic alterations in CRC. They found that 16% of CRCs were hypermutated: three-quarters of these had the expected high microsatellite instability, usually with hypermethylation and *hMLH1* silencing, and one-quarter had somatic mismatch-repair gene and polymerase e (*POLE*) mutations. In other studies, integrative analysis suggested an important role for MYC-directed transcriptional activation and repression. Epigenetic modifications like altered methylation events and modifications involving small non-coding RNAs have all been described in the pathogenesis of colorectal cancer (Ahuja et al., 1998; van Engeland et al., 2011; Jones et al., 2015).

The extensive complexity of colorectal cancer, and indeed cancer in general, requires unconventional methods do decipher it. Integrative approaches are essential to understand the interaction between genetic, epigenetic and otherwise non-genetic factors in colorectal cancer pathogenesis, including the application of system biology and network analysis into genomic data (Kreeger and Lauffenburger, 2010; Zhang et al., 2013; Emmert-Streib et al., 2014). Yu et al. (2015) studied the modules of protein–protein interaction (PPI) networks of differentially expressed genes. Functional enrichment analysis showed that differentially expressed genes involved in these modules were mainly associated with cellular activities. Shi et al. (2012) investigated integrated information from network, expression, and mutation data and demonstrated that the network-based data integration method provides a convergence between biological relevance and clinical usefulness in gene signature development. Next generation sequencing is promoting such network and pathway analysis to add much more to our understanding of tumors biology.

We describe here the results of a family based study of the genetic alterations in patients with familial colorectal cancer compatible with autosomal dominant pattern of inheritance, compared to healthy related controls. The aim was to delineate the differences in genetic mutation patterns between cases and controls using comparisons of mutational signatures and network analysis, in an effort to explain the predisposition to cancer development, to describe the genetic pathogenesis and to facilitate genetic counseling. We used Whole Exome Sequencing with subsequent comparisons of mutated genes between cases and controls followed by gene set enrichment and network analysis. The findings revealed an interesting mutational pattern in tumor tissues reflected by enrichment of genes sets affected by mutations – especially high impact short insertions–deletions – in tumor tissues for certain pathways and GO terms, a feature that was clearly less prominent in controls.

## Results

Here, we present the results of comparisons of genetic alterations and mutational signatures between cases and controls, followed by the results of enrichment analysis. The striking feature in this family is the issue of identical by state (IBS) sharing of variants observed in the cancer tissues as shown below, where comparison of cases to controls did not conform to the expected identical by descent (IBD) sharing especially among siblings. In accordance with culture in this part of the country, consanguinity is common but an *F*-test would be inconclusive given the comparison is between blood and tissue samples with the latter being under higher mutational load, manifested in tumors often having more IBS sharing as discussed below. The lack of germline whole exome sequencing for the two cases is a shortcoming we sought to circumvent by adopting an indirect inference approach for identity testing. BED files of variants from tumor tissues and controls are available on Galaxy Main Instance (https://usegalaxy.org/u/iend/h/colonexome) or otherwise upon request from the corresponding author.

### Patient Recruitment and Phenotyping

**FIGURE 1:**
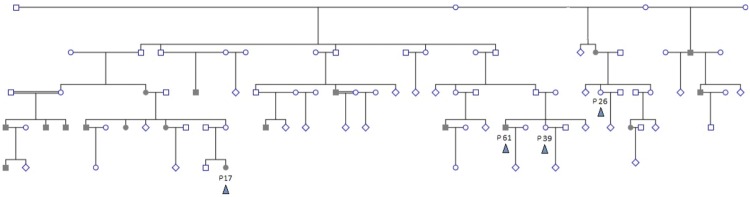
**Extended family pedigree showing sampled individuals and their relations.** Four sampled individuals (colored arrowhead) are shown – patients P17 and P61, and controls P39 and P26. P84 is distantly related and not shown. Shaded individuals are diagnosed with colorectal cancer. Decease status is not shown. Double-lines indicate second degree consanguinous marriage. Multiple healthy siblings and/or branches with no affected offspring are depicted as diamonds for simplification.

The study examined whole exome sequences of samples from five subjects chosen from a Sudanese family with a strong family history of hereditary colorectal cancer (as well as other tumors and diseases) by convenience non-probability sample selection. Two patients (male/female) were selected from two different branches of the extended family. Cancer patients had a histopathological diagnosis of Microsatellite Instability-High moderately differentiated colorectal adenocarcinoma. A diagnosis of hereditary non-polyposis colorectal cancer (HNPCC) was made based on Bethesda Criteria. Three controls were selected to comprise a sibling to one patient, a second closely related individual, and a third distantly related control. The family pedigree is shown in **Figure 1**. Tumor tissue samples are labeled P17 and P61, and controls are labeled P39, P26, and P84. The relations between individuals are shown in the pedigree (**Figure 1**). The family of individuals P39, P26, P61, and P17 is related to a common grandparent. Individual P84 is a distant relative of the family and hence not shown in the pedigree.

### Exploratory Analysis for Mutational Signatures

**FIGURE 2:**
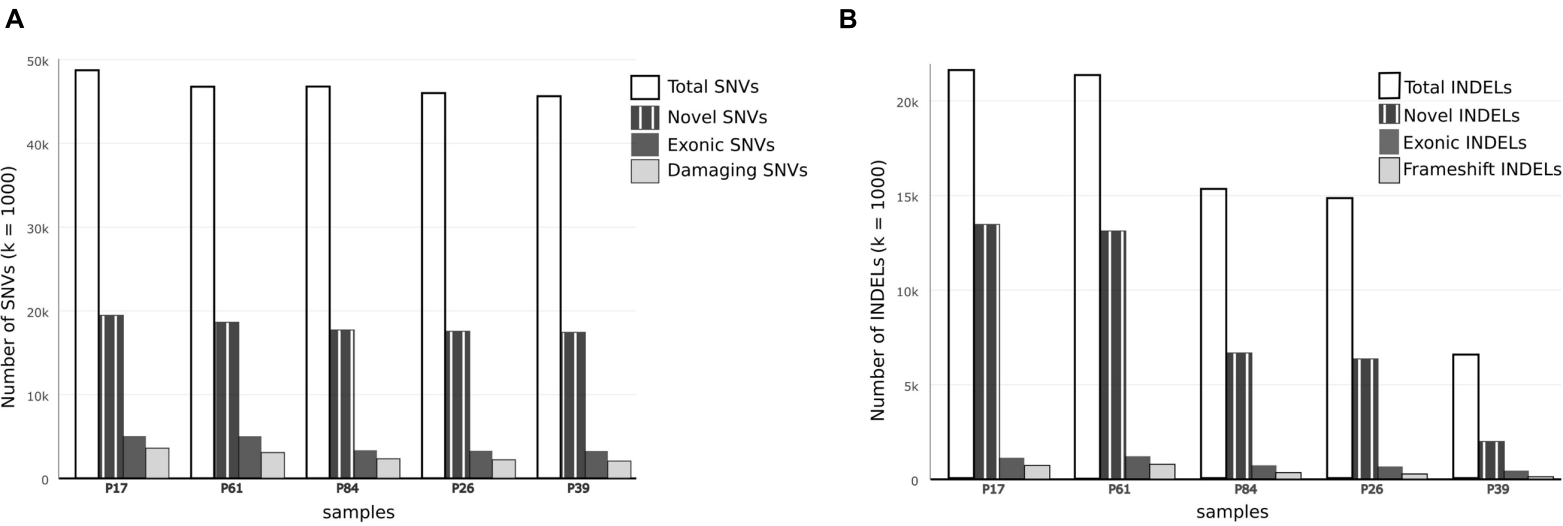
**Comparison of single nucleotide variations and insertion–deletions in study samples. (A)** Bar chart showing Total number of SNVs, number of damaging SNVs (assessed by ConDel scores) and novel SNVs (not found in 1000 genome) in study samples. **(B)** Bar chart showing total number of INDELs and number of novel variants with reference to dbSNP137. Number of exonic and frameshift INDELs is shown as well. The discrepancy between tumor and control samples is more prominent in INDELs.

**FIGURE 3:**
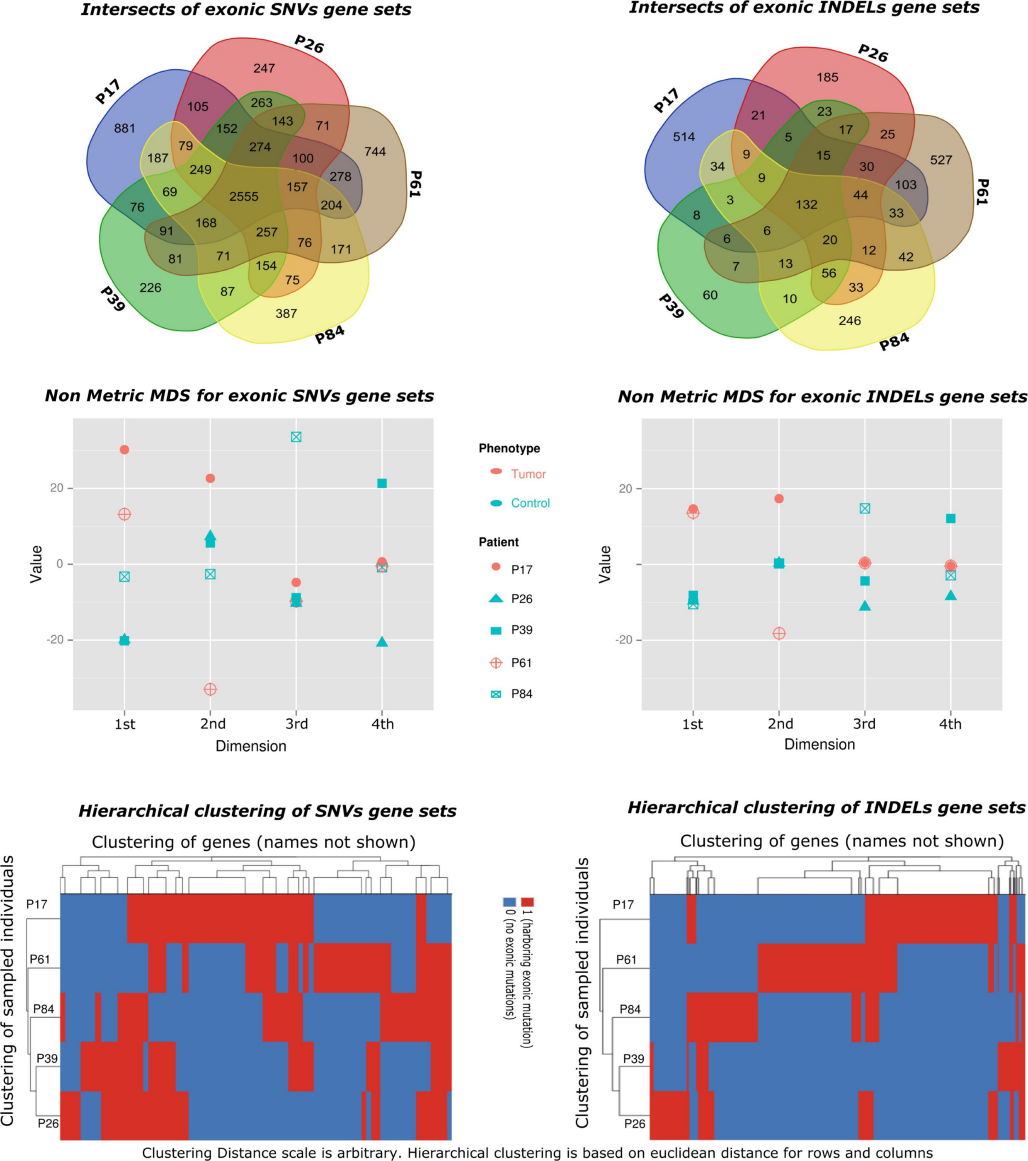
**Gene set-based exploratory analysis. (Top)** Pattern of affected gene sharing between samples is summarized as intersections of SNVs/INDELs gene sets (Venn diagrams). Gene sets for INDELs included genes harboring splice site or exonic insertions–deletions. Gene sets for SNVs included genes harboring splice site or exonic (stop gain, stop loss, or non-synonymous) changes. **(Middle)** Non-Metric Multidimensional Scaling of distance matrix between samples based on INDELs and SNVs gene sets (set of genes affected by exonic INDELs and SNVs, respectively). The matrix was constructed using affected genes as variables (columns) and sampled individuals as rows. A gene is assigned a score of one if it showed an exonic or splice site change, or a zero otherwise. Non-metric multidimensional scaling is shown in four dimensions (maximum number of dimensions = N-1, where N is the number of rows). The stress value is <0.1 (approximates zero). Tumor samples are shown in red. Control samples are shown in blue. The clustering of tumor samples is evident in the first dimension especially for INDELs. **(Bottom)** Hierarchical clustering of samples using the same distance matrix described above is depicted and reflects separation of cases from controls. Clustering distance is larger between tumors and controls (rows) for INDELs compared to SNVs.

There were higher numbers of variants in tumor samples (**Table 1** and **Figure 2**). We utilized unsupervised learning to find hidden structure in unlabeled data (blinding the phenotypes) using gene-based approach (genes affected by exonic and/or splice site SNPs/INDELs used as variables; details in methods summary). We used non-metric multidimensional scaling in four dimensions to look for a pattern of gene affection by exonic variants between the five samples (**Figure 3**). When we studied INDELs, the first dimension completely separated cases from controls with tight sub-clustering of tumor and control samples; the second dimension perfectly separated the two cases while the third and fourth dimensions reflected the variability between the controls (**Figure 3**). The fact that multiple dimensions provided complete separation of cases and controls – without including the phenotypes in the clustering matrix – strongly implies that the observed pattern of gene hits in INDELs is significantly deviated from simple randomness. Hierarchical clustering of the distance matrix provided comparable results. When analyzing SNVs, clustering of controls was less evident (**Figure 3**). Except for the third dimension, which likely reflected IBD sharing of alleles resulting in a separation pattern of samples comparable to the pedigree, P84 showed a behavior similar yet intermediary to cancer samples in the first and second dimensions, deviating from expected clustering with the two other controls. We can argue from this preliminary analysis that a gene-based signature of exonic INDEL hits is a reasonable possibility in tumorigenesis. However, a distinct pattern in each tumor is clearly seen as well.

**Table 1:** Number of single nucleotide variations and insertions–deletions per study sample and number of variants in each class.

		Variations count						
Class	Sample	All	Novel	Exonic	Intronic	UTR3	UTR5	Splicing
SNVs	P17	48731	5097	19509	21904	2129	1041	281
	P61	46761	5078	18682	20227	2019	1083	267
	P84	46781	3402	17768	22041	2072	971	264
	P26	46000	3332	17611	21372	2101	1013	254
	P39	45627	3310	17477	21148	2082	1037	133
INDELs	P17	21566	13473	1080	16129	1221	288	65
	P61	21330	13130	1174	15762	1136	332	76
	P84	15284	6682	661	11008	850	231	84
	P26	14797	6367	604	11043	851	223	78
	P39	6530	2004	411	4090	339	102	49

*P17 and P61 are tumor tissue samples. P39, P26, and P84 are peripheral blood control samples. SNVs, single nucleotide variations; INDELs, insertions–deletions, UTR, untranslated region.*

### Comparison of SNVs and INDELs in Tumor Samples and Controls

In both tumor samples, *hMSH2* loss of function truncating mutation (rs63749917) was found, consistent with Lynch syndrome. Although generally there were higher numbers of variants (especially insertions–deletions, referred to here as INDELs) in tumor samples (**Table 1**), the number of non-synonymous mutations was consistent with figures from other studies in accordance with evolutionary conservation of the functional protein-coding part of the genome and the higher mutation rate of neutral non-coding regions. However, the overall number of damaging variants in the tumor tissues (as identified by ConDel scores, see Methods) was significantly higher with a stepwise increase in damaging mutations. Sample P39 showed the lowest amount of damaging SNVs (hence considered here as a true control) and tumor sample P17 exhibited the highest number while control sample P84 was located in an intermediary position (**Figure 2**). The number of INDELs was also highest in the two tissue samples and displayed a unique feature of ordered mutation pattern shown below in GO enrichment. Although two controls (P26, P84) have shown similar features of possible genome instability – indicated by number of INDELs – comparable to those in tumor samples (**Figure 2**), there was no orderly pattern of enrichment in the INDELs gene sets. Besides, none of them developed symptoms of cancer over the past decade, indicating again that the pattern of mutations is a necessary feature of cancer development and pathogenesis.

An interesting feature seen in this family is the sharing of excess variants more than expected by IBD for tumor tissues. Unsupervised clustering of alleles in all variation positions showed deviation from expected allele sharing with a clear separation of tumor samples from control that did not reflect the relations perceived from the pedigree. The two tumors shared more variants than what is expected from familial allele sharing, while the difference between closely related individuals, for instance between the two siblings, patient P61 and control P39, was large. The explanation of this sharing is cumbersome and one of the few possible mechanisms for such ordered allele change is DNA editing and site directed DNA transposition. DNA and RNA editing are ancient mechanisms that originated possibly as early as the emergence of Euokarya. We investigated the possibility of editing which was recently reported to occur in cancer tissues through members of the APOBEC family of enzymes (Gallois-Montbrun et al., 2007; Roberts et al., 2013) by comparing the percentage of A–G and C–T transitions. We examined novel SNPs and those of low allele frequency or otherwise not reported in the 1000 Genome database as these are likely to be a product of editing. The observed percentages conformed to editing ratios by excess of A–G/C–T transitions, constituting 80–85% of the entire substitutions in the novel SNVs compared to an average of 66–70% in known SNPs (Fisher *p*-value 5e-6). To test the reliability of this finding, we compared A–G and C–T transitions ratios in inferred IBS somatic variants (described below) which revealed excess in A/G and C/T ratios as well (**Table 2**).

**Table 2:** Transitions and transversions in inferred identical by state somatic SNVs.

	Alt. Allele			
Ref. Allele	A	C	T	G
A	–	38	20	149
C	43	–	158	63
T	32	169	–	38
G	220	63	47	–

*The table shows A–G and C–T transitions in inferred IBS variants (shared variants between tumor samples replicated in TCGA). Ref, reference; Alt, alternative.*

### Identity by State Inference

It is difficult to identify IBS with precision in a small number of samples in a consanguineous family as we argued. However, we adopted a unique inference approach based on simple comparison between tumor samples, control samples, and TCGA Colonic Adenocarcinoma somatic mutations (see Methods) to identify variations with a high likelihood of being IBS between tumor samples. The concept was based on identifying variations present in both tumor samples and at least one TCGA sample (considered a positive control) provided that the variations are not seen in any of our controls (negative controls). We identified slightly more than 1100 variations, very likely to be somatic IBS variants between tumor samples, corresponding roughly to more than 900 genes (Supplementary Data: Data Sheet 1). Many of these genes were related to cancer. For instance *POLE* was in this set of genes along with *POLE4, POLD1, POLI, POLQ*, and *REV1*. This set of genes showed a dense interaction network with multiple protein–RNA interactions centered on *ELAVL1/HuR*, and remarkable patterns of enrichment. Particularly, some miRNA (mir377, mir199, and mir486), transcription factors (e.g., NFKB1, NFE2), GO terms [extracellular matrix (ECM) organization, collagen metabolism, DNA binding] and pathways [ECM Receptor Interaction (KEGG), ECM Organization (Reactome)] showed significant enrichment. We compared A–G and C–T transitions ratios in inferred IBS variants. This subset of variants showed very little number of novel variations but reserved the excess in A/G and C/T ratios (**Table 2**).

### Cancer Signature

Although cancer is a multistep complex process, Mendelian-like cancer syndromes are thought to be caused by a limited set of tumor suppressor genes (TSG) germ line mutations. In both tumor (and sometimes control) samples, there was a number of mutations in known cancer-related genes. We verified cancer mutational signatures through gene ontology enrichment and network analysis. We used GO term enrichment, pathway enrichment, and interaction network analysis to identify the critical genetic alterations involved in the tumorigenesis process as well as to highlight possible genetic circuits of predisposition.

### Pathway and GO Enrichment

Tested against KEGG database, enriched pathways unique to the tumors included ABC transporters, Lysine degradation, MODY (maturity-onset diabetes of the young), Pantothenate and CoA biosynthesis, cell cycle, and cell adhesion as well as olfactory receptors (**Table 3**). Individually, patients and controls showed variable enrichment patterns. Yet in the cancer samples, ECM receptor interaction, ABC transporter, and olfactory transduction were maintained, while controls P84, and P26 showed enrichment in ECM-receptor interaction and glycosaminoglycan degradation respectively. Control P39 had no significant enrichment in any pathway. The Wnt/B-catenin is an important pathway in cancer and particularly associated with colorectal tumors. Mutations may dictate not only the prognosis, and type of cancers in an individuals but population profiles as well. Binding of LRP and FZ in the canonical Wnt pathway disrupts the destruction complex for catenin degradation and thus mutations in these genes (as in tumor sample P17) would cause it to accumulate along with the mutations in destruction complex pathway. Sample P61 showed a *CTNNB1* mutation that has been reported to cause the same accumulation leading to cancer. RNF43 mutations were encountered in both cases while *DVK1* and *FZD8* are mutated in P61. Individual mutations in *AXIN2* and *CSNKIE* in sample P17 may similarly contribute to such effects. Enrichment in the olfactory receptor family, which was seen, is probably not due to the size of the gene family as claimed by some authors (Glusman et al., 2001; Niimura and Nei, 2007; Young et al., 2008), as the damaged genes turned to be over-represented compared to other members of equally large gene families like the histone and actin, which were all underrepresented in the exome data set (*p*-value: 2e-4) particularly in the non-synonymous mutations.

**Table 3:** Enriched pathways and their *p*-values in mutated gene sets in our study samples.

Pathway	P17	P61	P26	P39	P84
ABC transporters	0.017	2.1e-06	–	–	–
ECM-receptor interaction	0.017	0.0064	–	–	0.0031
Focal adhesion	–	–	–	–	0.0362
Glycosaminoglycan degradation	–	0.0064	0.00075	–	–
Lysosome	–	0.035	–	–	–
Metabolic pathways	0.043	–	–	–	–
Olfactory transduction	3.7e-12	4.1e-07	–	–	–
Pantothenate and CoA biosynthesis	–	0.0015	–	–	–

The greatest discrepancy in GO enrichment was seen in INDELs-affected gene sets. Cancer samples showed significant enrichment of certain GO terms. On the contrary, all control samples did not feature any significant enrichment for INDELs-affected gene sets. In line with our previous assumption on the importance of INDELs pattern, none of the controls showed significant over-represented GO terms; in fact, control 26 showed statistically non-significant over-representation of few terms, while P84 and P39 showed no over-representation at all. On the other hand, the two cancer cases showed significant over-representation of DNA binding proteins and signaling proteins (Supplementary Data: Image 1). This may further supplement the evidence that INDEL pattern is not at all random in colorectal cancer tissues.

### Network Analysis

**FIGURE 4:**
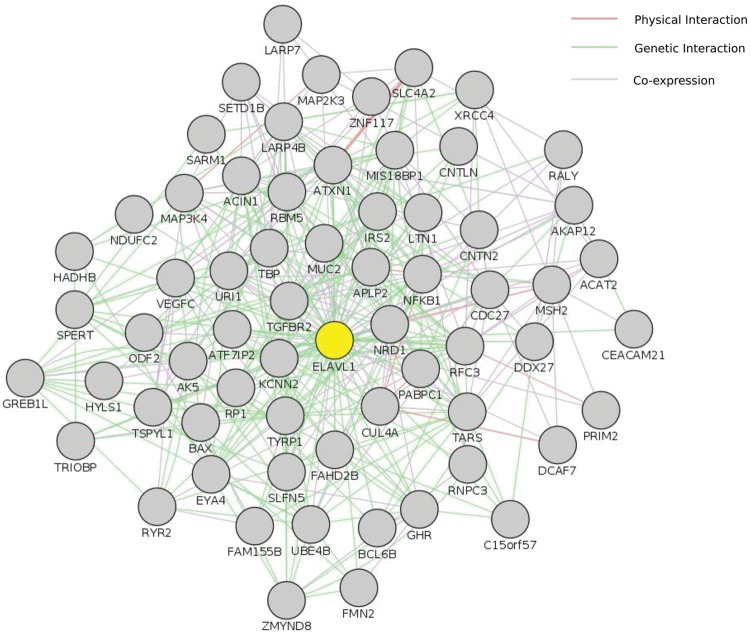
**Centrality of ELAVL1/HuR.** This figure shows ELAVL1/HuR direct interaction network with genes harboring high impact genetic alterations in both tumor samples in this study. Input included perturbed genes harboring damaging genetic changes (both SNVs and INDELs). Only genes shared by both tumor samples were included. This interaction network was constructed using GeneMania cytoscape app. ELAVL1 is highlighted in yellow. The complete set of ELAVL1 interactions is also available as supplementary Image 2.

We used two different interaction databases to study our gene sets (Reactome and VanBuren Lab Cognoscente). To study the genetic and protein interactions of mutated genes (genes harboring high impact or damaging mutations) with other molecules (including Protein–Protein, Protein–RNA, and Protein–DNA interactions), we used VanBuren Lab online tool Cognoscente that draws genetic interaction networks between input genes and also highlights potential genetic interactions with other intermediates. The advantage of such network approach is the insights it gives into the potential biological mechanisms involved that are not based directly on mutations within the gene sets. We used all genes affected by exonic or splice site variants (INDELs and damaging SNPs defined by ConDel scores) in both tumor samples as input. The most spectacular feature was the interaction of SNVs and INDELs with the RNA-binding protein *ELAVL1/HuR*, a hub protein of remarkable centrality interacting with over 2000 proteins in the human proteome, together with another central hubs: *UBC, TP73*, and *NFKB1* (Supplementary Data: Image 2). The direct interactions between ELAVL1 and mutated genes in tumor samples are shown in (**Figure 4**). Although *ELAVL1/HuR* and *UBC* are not mutated in our samples, they were extensively interacting with a large number of input genes. To address whether such interaction particularly the centrality of the *ELAVL1/HuR* is an artifact of the large interacting partners or true interactions we compared the percentage of interactions in genes having non-synonymous SNVs and genes with synonymous SNVs to test the randomness of interactions. The synonymous set had no significant interactions and ELAVL1/HuR was interacting with only less than five nodes (Supplementary Data: Image 3). We queried The Cancer Genome Atlas database for case records with comparable clinical and pathological phenotypes to our studied samples (family history of colorectal cancer and loss of *hMSH2* expression). We looked at exonic somatic mutation data for four patients we could identify (TCGA IDs: TCGA-CM-5864, TCGA-CM-6674, TCGA-CM-6164, and TCGA-AY-6196). In two cases, ELAVL1/HuR had relatively few interactions. Instead, another hub protein, TP53, showed marked centrality (Supplementary Data: Images 4–7).

**FIGURE 5:**
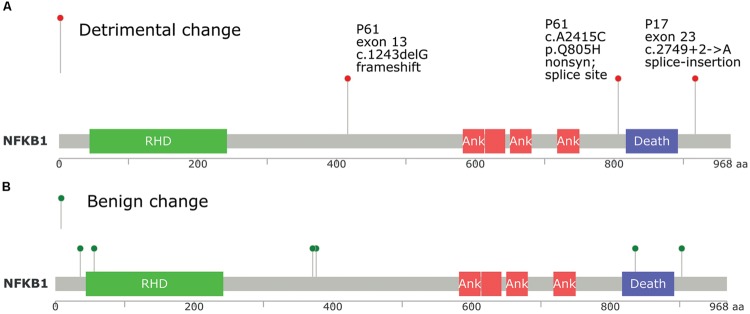
**Position of *NFkB1* mutations found in tumor samples P17 and P61 compared to TCGA database, and its relation to protein domains. (A)** *NFkB1* mutations in this study. **(B)** *NFkB1* mutations in TCGA queried using cbioportal.org. RHD, Rel homology DNA binding domain; ANK, ankyrin repeat domain; Death, death domain.

We utilized a different approach to draw only direct PPI networks for genes affected by all (not only damaging) exonic/splice site variants, for each sample, based on Reactome database. Network centrality was investigated using three different methods of un-weighted centrality (degree, betweenness, and closeness centrality). In tumor samples, we found that *NFKB1, HDAC2, PIK3R1, TCF7L2, ITGAV, TRAF2*, and *CDC27* were top central nodes in sample P17, while in sample P61, *NFKB1, ACTN2, PPP2R1B, RELA, PIK3CB, SIRT1*, and *CAMK2B* were central nodes. Overlap of interaction networks of tumor samples showed an interesting feature of increased sharing around central nodes (Supplementary Data: Image 8). As well, clusters of related genes (e.g., receptor variants) were also prominent. *NFKB1, TCF7L2, TGFBR2, NCOR2, CDC27, ACTN2, PIK3R1/2, PPP2R1B/2A, PABPC1, TBP* along with different *MAPK* and *STAT* proteins were centrally shared. *NFkB1* was the most central protein in direct PPI network, and both cases in our study had NFkB1 mutations (**Figure 5**). Bearing in mind the intricacies of NFkB interactions, it is almost impossible to predict the change in the signaling pathways based on mutational testing alone. However, the centrality of NFkB1 in the interaction networks of mutated genes is not something to overlook.

### Predisposition Circuitry

The potential existence of predisposition circuitries is highlighted by two findings: firstly by the network of genetic interactions of genes shared between cancer samples (P17, P61) and controls (P39, P26, and P84) where the gene ELAVL1/HuR was central together with candidate cancer genes like *WHSC1, ERBB2, PRNP, PABPC1, TNFRSF14, MAP2K3*, and *UBC*; and secondly the evolving complexity of interaction networks from control to tumor samples (Supplementary Data: Image 8) which is consistent with the preliminary assumptions we made on a stepwise pattern of predisposition and ordered mutations. The number of PPI network nodes and edges was higher in colorectal cancer tumor samples. The percentage of genes forming a single major functional interaction network (using Reactome database) in samples P39, P26, P84, P17, and P61 out of the total number of gene sets was 4, 17, 19, 26, and 29%, respectively. This was visually detectable from the topography of interaction networks (Supplementary Data: Image 8).

## Discussion

Cancer is a group of diseases characterized by multiple phenotypes including abnormal cell growth and the potential to invade or spread to other parts of the body, reflecting the complexity of the biological processes involved. Given the plethora of non-synonymous mutations leading to damaged or altered protein functions in healthy individuals, cancer seems like a probabilistic combination of events targeting the biological processes, that raises the fitness of tumor cells. The question “do somatic events occur randomly or due to ordered mutagenesis?” remains unsolved. The network approach employed in the current analysis combined with an individualized and familial context fostered insight into the stepwise and multiple stages of the carcinogenesis process, and allowed addressing these broad themes of predisposition circuitries and mutation randomness. Particularly, inclusion of familial proband controls allowed inferences on the potential role of germline genetic alteration in pathway and network analysis, compelling a revisit to the concept of pathognomonic cancer mutations in disease predisposition. Hundreds of driver mutations were identified following the introduction of exome sequencing studies. For example, 558 interactions and 717 regulatory connections existed among 163 known cancer genes and 164 predicted driver genes extracted from the database DAVID (Chen et al., 2013a). March et al. (2011) reported in a mouse model and a comparison to human data sets that 234 genes are dysregulated in colorectal cancer. Of those 70 genes showed the pairwise co-occurrence clustering into 38 sub-networks that may regulate tumor developments (March et al., 2011). Most exome investigations focused on analyzing cancer cases and the role of somatic mutations, with no or little insight on the role of germ line mutations. Few studies questioned the role of germ line mutations. One study analyzed 35 potentially functional single-nucleotide polymorphisms in 10 candidate cancer genes and based on the functional relevance suggested that no influence of the studied germline variants existed on CRC risk and survival (Huhn et al., 2014). The recent demonstration that genes/proteins of centrality are prone to contrasting modes of natural selection as opposed to peripheral ones (Luisi et al., 2015), emphasizes the significance of the network as an evolutionary unit of inheritance, and the role of these central genes in health and diseases including cancer.

The current set of data was used to explore how such molecular events may contribute to the carcinogenesis process in an attempt to shed light on the actual complex etiology of the disease from a network rather than a single gene perspective. One challenging question is the magnitude and threshold of accumulating somatic mutations that will tip off the homeostatic balance toward cancer, bearing in mind the obvious existence of predisposition circuitries among cancer-free controls. Variants were not just in numerical excess in the tumors but targeted fundamental pathways and genes in the cancer process including cellular metabolism, cell cycle, angiogenesis, cell adhesion, and DNA repair: in short all processes required for cell survival and fitness.

As both germline and somatic point mutations including splicing are rather conserved given their reported mutation rate, they are unlikely to explain all the biological alterations leading to function in cancer. This renders RNA and DNA editing – both rather uncommon events in the human genome – along with insertions–deletions, likely and plausible culprits of such alterations. These events, however, whether affecting genes or protein networks, seem to be least random in any sense, specially with the notable sharing of variants between the two tumors. Such compelling display of ordered mutagenesis suggests an orchestrating molecule or a mechanism that governs or at least sets the action of the network on course and may explain the shared mutation pattern encountered in tumors. This molecule is envisioned to be a central part of the potential cancer cell. The milieu of this cell functions as a system with an input signal (coupling this molecule to initiate the perturbation of the cell through complex signaling processes) and converging on complex output signals that dictate the phenotype. The input signal is explanatory to the nature of the perturbation and how the circuits and cascade exhibited by the cancer cell are switched on. The most plausible candidate for the input signals, as we will argue, are oncogenic viruses and primarily in our case Epstein–Barr virus (EBV). A viral infection is envisioned to interact with predisposition networks which include counterpart proteins in various downstream stages (including in the current data set: FOS, ELAVL1, NFkB1, POLR3B, and others at different cellular compartments and stages of the tumorigenesis).

The protein ELAVL1/HuR seems the most plausible candidate for coupling the initial viral input signal, yielding the expected effector and orchestrating role of the system given the repertoire of its interacting partners. ELAVL1/HuR is a hub protein like TP3 and TGF-Beta and is thought to be central and key in the carcinogenesis process. Interestingly TP53 was not implicated in the analysis either directly or indirectly, in contrast to examples from TCGA (Supplementary Data: Images 4–7), and consistent with previous literature on its minor role in breast cancer in this part of the world (Masri et al., 2002). The lack of TP53 mutations and absence of network centrality should receive some further inquiry. The role of ELAVL1/HuR in cancer has been reviewed by Wang et al. (2013) and discussed by Winkler et al. (2014) and López de Silanes et al. (2005). ELAVL1/HuR is enriched in the nuclear matrix fraction and linked to the regulation of colon adenoma to carcinoma progression (Albrethsen et al., 2010). Yiakouvaki et al. (2012) suggested a role for HuR as a homeostatic coordinator to guide innate inflammatory effects and linked it to pathologic inflammation and cancer. In colon cancer, HuR was shown to be increasingly expressed in the cytoplasmic epithelial compartment in consecutive stages of the adenoma-carcinoma sequence in FAP (Brosens et al., 2008). Also, COX-2 levels correlate with cytoplasmic expression of HuR in colorectal cancer specimens (Brosens et al., 2008; Lim et al., 2009). In fact, ELAVL1/HuR has been linked to both promotion and suppression of tumorigenesis (Shultz and Chalfant, 2012). The intracellular location of HuR likely affects the expression of a number of genes that are responsible for the invasive phenotype of CRC, making inhibitors of HuR possible anticancer drugs (Ignatenko and Gerner, 2008).

The significance of ELAVL1, which in itself is quite conserved and unaffected by direct genetic alterations, lies in its potential role in combination with other molecules. The role it plays along with viral oncogenesis (that is believed to be central to cancer etiology in our cohorts; Yahia et al., 2014) is supported not only by the extensive connectivity of the protein, but also its potential functional role in RNA/DNA editing. Editing is thought to be a pivotal contributor to carcinogenesis in this study and other recent literature (Paz et al., 2007; O’Bryan et al., 2013; Wang et al., 2013; Winkler et al., 2014; Chou et al., 2015). The main editing enzymes recognized in mammals are members of the APOBEC family – and namely in this case cytidine deaminase. Activation-induced cytidine deaminase (AID) is known to interact with ELAVL1/HuR and is a potential DNA/RNA editing enzyme (Muramatsu et al., 2000; O’Bryan et al., 2013). AID is responsible for the induction of three reactions of DNA somatic modification employed by jawed vertebrates in the context of adaptive immunity: somatic hypermutation (SHM), class switch recombination (CSR), and immunoglobulin gene conversion (Ig GC; Muramatsu et al., 2000). However, in conditions of dysregulation, AID has also been implicated in both lymphoid and non-lymphoid neoplasia (Koduru, 2012; Robbiani et al., 2015). ELAVL1/HuR was also implicated in DNA global epigenetic regulation, another feature of the viral etiology in relation to carcinogenesis, i.e., inactivation and modulation of major circuitries involved in carcinogenesis (Zhou et al., 2011). The cell reprogramming may account not only for the activation, silencing, and regulation of key cancer genes but also for the stem cell link in cancer and may attribute to the ability of cancer cell to perform multiple functions confined to differentiated cells, and the regression to primitive and unicellular behavior. In an independent study targeting the methylome of Sudanese breast cancer patients (Abdallah et al., manuscript in preparation), developmental pathways and EBV came out prominently, both highly enriched, conforming to our original hypothesis of EBV etiology of cancer through viral dysregulation of the methylome.

Wnt/B-catenin is an example of several intermediate signaling cascades that are involved in both development and carcinogenesis and in colorectal cancer is believed to be critical in shaping the tissue specificity of the tumor (Kolligs et al., 2002; MacDonald et al., 2009; Wallmen et al., 2012). In the current study, the individualized context was reflected in the fact that SNVs and INDELs were often unique in targeting genes or gene regions, but converging in dysregulation of the pathway as shown in the result section. Wnt/B-catenin couples several molecules and networks, including ELAVL1/HuR and NFkB (proteins that we propose for an input and a downstream output signal, respectively; Kim et al., 2012a,b).

Experimental evidence for NFkB role in cancer was demonstrated. Djavaheri-Mergny et al. (2007) observed that TNF-alpha induced NFkB activation causes repression of autophagy. It has been found that the temporal pattern of NFkB activation influences apoptotic cell fate in a stimuli-dependent fashion (Fan et al., 2002). As well, mutations of NFKB1 gene, the precursor of p50, are observed in many solid tumors (Rayet and Gélinas, 1999; Karin, 2009; Jiao et al., 2012; Hoesel and Schmid, 2013). Moorchung et al. (2014) showed that constitutive activation of NFκB was frequently observed in CRC. A polymorphism in NFkB was associated with CRC risk in Danish population, suggesting a role for NFkB in CRC etiology (Andersen et al., 2010). Another study indicated that the anti-inflammatory and antitumor activities of Gallotannin, a plant polyphenolic compound, may be mediated in part through the suppression of NF-κB activation (Al-Halabi et al., 2011). The classical proposed mechanism for NFKB role in colorectal and other solid tumors is constitutive activation via defective regulatory proteins activity rather than changes in NFKB1 gene (Courtois and Gilmore, 2006). Both tumors and one control (P84) in our study, showed NFKB1 mutations, emphasizing the role of interactions and predisposition (mutation positions in each of the tumor samples are shown in **Figure 5**). The DNA binding domain did not show mutations. It is difficult to perceive why some malignant cells would break a central key to cellular functions like NFKB. Yang et al. (2011) data provide strong evidence that NFkB can function as a biphasic regulator either suppressing or enhancing ovarian cancer through the regulation of MAPK and cellular apoptosis. They showed that NFkB functions as a tumor suppressor in four ovarian cancer cell lines, but it functions as an oncogene in their aggressive chemo-resistant isogenic variants. In this sense, mutations in NFkB1 observed in many tumors might be selected as a mechanism for counteracting a pro-apoptotic effect in order to tip the balance toward anti-apoptotic environment.

## Conclusion

Exome sequencing of members of an extended family with colorectal cancer revealed interesting features of stepwise accumulation of mutations leading to tumorigenesis, a shared mutation pattern and predisposition circuitry. We highlighted the importance of central hub proteins in the study of cancer genomics and pathogenesis. RNA binding protein ELAVL1/HuR and NFKB, both of which appeared central in the network analysis, are suspected to work in concert with viral etiology on a background of predisposition circuitry to initiate and guide the pathogenesis of colorectal cancer in a subset of predisposed individuals.

## Methods

### Sample Preparation and DNA Extraction

DNA was extracted and purified from frozen colorectal tumor tissues (tumor samples) and EDTA-preserved blood (controls) using Wizard^®^ Genomic Purification Kit (Promega, USA). Tissue lysates and extraction solutions were prepared according to the Wizard^®^ SV Genomic DNA Purification System protocol. Extracted DNA quantity and quality were assessed using the NanoDrop^®^ ND-1000 (NanoDrop Products, Wilmington, DE, USA) spectrophotometry at wavelength spectrum of 220–750 nm and standard 1% agarose gel electrophoresis.

### Whole Exome Sequencing

Library preparation, Exome Capture, and Whole Exome Sequencing were performed at BGI^®^ (Hong Kong, China). Library preparation and exome capture were done using NimbleGen Oligonucleotide Libraries (Roche NimbleGen, USA). Exome sequencing was performed on Illumina HiSeq2000 platform (Illumina, USA). The clean reads (after removal of adapter sequence in the raw data, low-quality reads and reads with more than 10% unknown nucleotides) were aligned to the reference human genome build hg19 using Burrows–Wheeler Aligner (Li and Durbin, 2009). The average coverage in study samples ranged from 107 to 210x. Quality control (QC) was present in the whole pipeline. SNPs were called using SOAPsnp (Li et al., 2009). Following local realignment using GATK (McKenna et al., 2010), INDEL variant calling was performed using Atlas2 (Challis et al., 2012). Annotation of all variants was performed using ANNOVAR (Wang et al., 2010).

### Exploratory Analysis

Gene sets were prepared by filtering for all genes harboring exonic and/or splice site variations stratified into SNVs and INDELs gene sets for each sample. Distance matrices for each category were constructed using R *stats* package dist() function (R Core Team, 2014) by concatenating all genes with exonic variants and subsequently using a binary scoring matrix where an affected gene is given a mark of 1 and a spared gene is given a mark of zero. R function isoMDS in the package *MASS* (Venables and Ripley, 2002) was used for non-metric Multi-dimensional Scaling. Heatmap visualization of these distance matrices was done using R package pheatmap (Kolde, 2012).

### Pathway and GO Enrichment

EnrichNet network-based enrichment analysis online tools (Glaab et al., 2012) were used to test pathway enrichment for gene sets in multiple databases (KEGG, NCI, Reactome). Enrichr online query tool (Chen et al., 2013b) was used to test the enrichment pattern for inferred IBS gene sets. Cytoscape ReactomeFI (Wu et al., 2014) application provided pathway enrichment testing for gene sets in Reactome Protein–Protein Functional Interaction Networks. Cytoscape BiNGOapp (Maere et al., 2005) was used to visualize over-representation of GO terms. BiNGO maps the predominant functional themes of a given gene set in the GO hierarchy and outputs this mapping as a Cytoscape graph. Hypergeometric distribution, with FDR correction and ‘0.05’ significance was used as app setting for all gene sets.

### Network Analysis

INDELs and non-synonymous SNVs in Exonic/Splice sites were used in gene/protein set construction. Although other types of INDELs/SNVs (UTR, upstream, Intronic, downstream) have an influence on genomic milieu, they were not included in these shortlists for network construction due to the lack of consensus scoring criteria to classify their effect. The goal was to reduce false positives as much as possible. VanBuren Lab online tool Cognoscente (VanBuren and Chen, 2012) was used for expanded genetic network construction for DNA–RNA–protein interactions for gene lists. When needed, damaging SNVs were defined according to Condel/FannsDB scores (González-Pérez and López-Bigas, 2011). In this case, centrality of genes was conceived visually from the topography of the network.

Cytoscape ReactomeFI App was used to construct individualized functional interaction networks for gene sets from each sample. In each sample, we used the network that included the largest possible number of genes for further visualization and centrality analysis (excluding small networks of gene clusters – usually less than 10–15 genes). Cytoscape RINalyzer app (Doncheva et al., 2011) was used for pair-wise network comparisons and overlap. Cytoscape GeneMANIA app was used to draw direct ELAVL1 interactions in **Figure 4** (Warde-Farley et al., 2010). Cytoscape CytoNCA app (Tang et al., 2014) was used for un-weighed centrality (degree, betweenness, closeness) ranking. Cytoscape (Shannon et al., 2003) workflow tool was used to create centrality based custom styles.

### TCGA Queries

Online repositories for TCGA data available on TCGA data portal (Hudson et al., 2010) and cbioportal (Cerami et al., 2012) were used to access TCGA records.

## Ethics Statement

This study was ethically approved by the Ethical and Scientific Committee of the Institute of Endemic Diseases, University of Khartoum. Written informed consent was obtained from all participants, in accordance with the Declaration of Helsinki.

## Author Contributions

SS, MI, OE, HM, MR, LB, FB, AS, MN: conceived the study and the experimental design. MK, WN, MA, UE, AH, OE, SF, FI, KA, MI: designed and carried out the analysis. All authors participated in interpretation of data. MI, MK, WN, wrote the paper. SS and MK contributed equally to this work. All authors revised the manuscript critically and were involved in editing the paper and approval of the final manuscript.

## Conflict of Interest Statement

The authors declare that the research was conducted in the absence of any commercial or financial relationships that could be construed as a potential conflict of interest.
