# Supplementary figures and images for: Exome sequencing of a colorectal cancer family reveals shared mutation pattern and predisposition circuitry along tumor pathways
Source: Front Genet. 2015 Sep 15;6:288. doi: 10.3389/fgene.2015.00288 (PMC4584935; doi:10.3389/fgene.2015.00288)

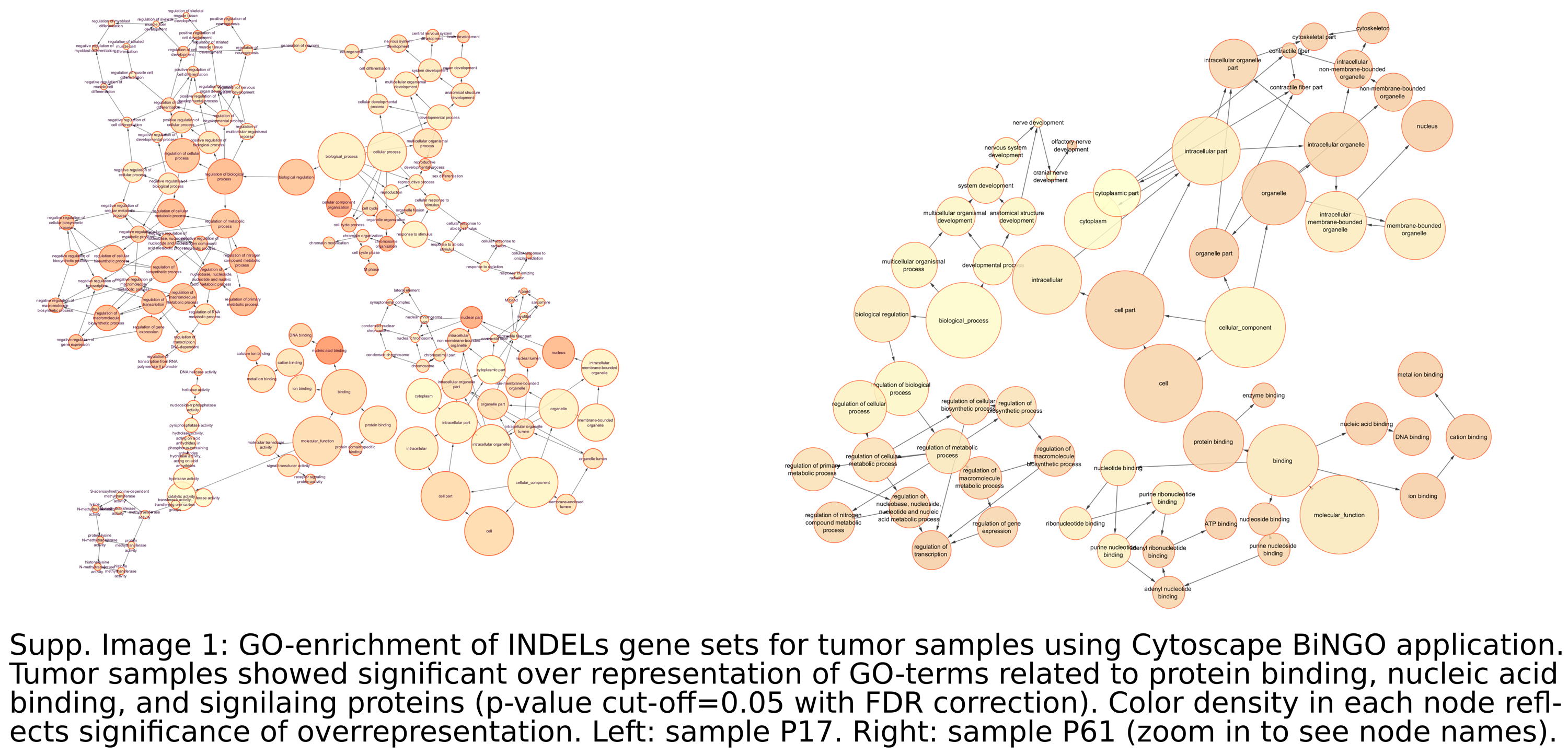

Supplement: Supplementary file 2 [file Image_1.PNG]

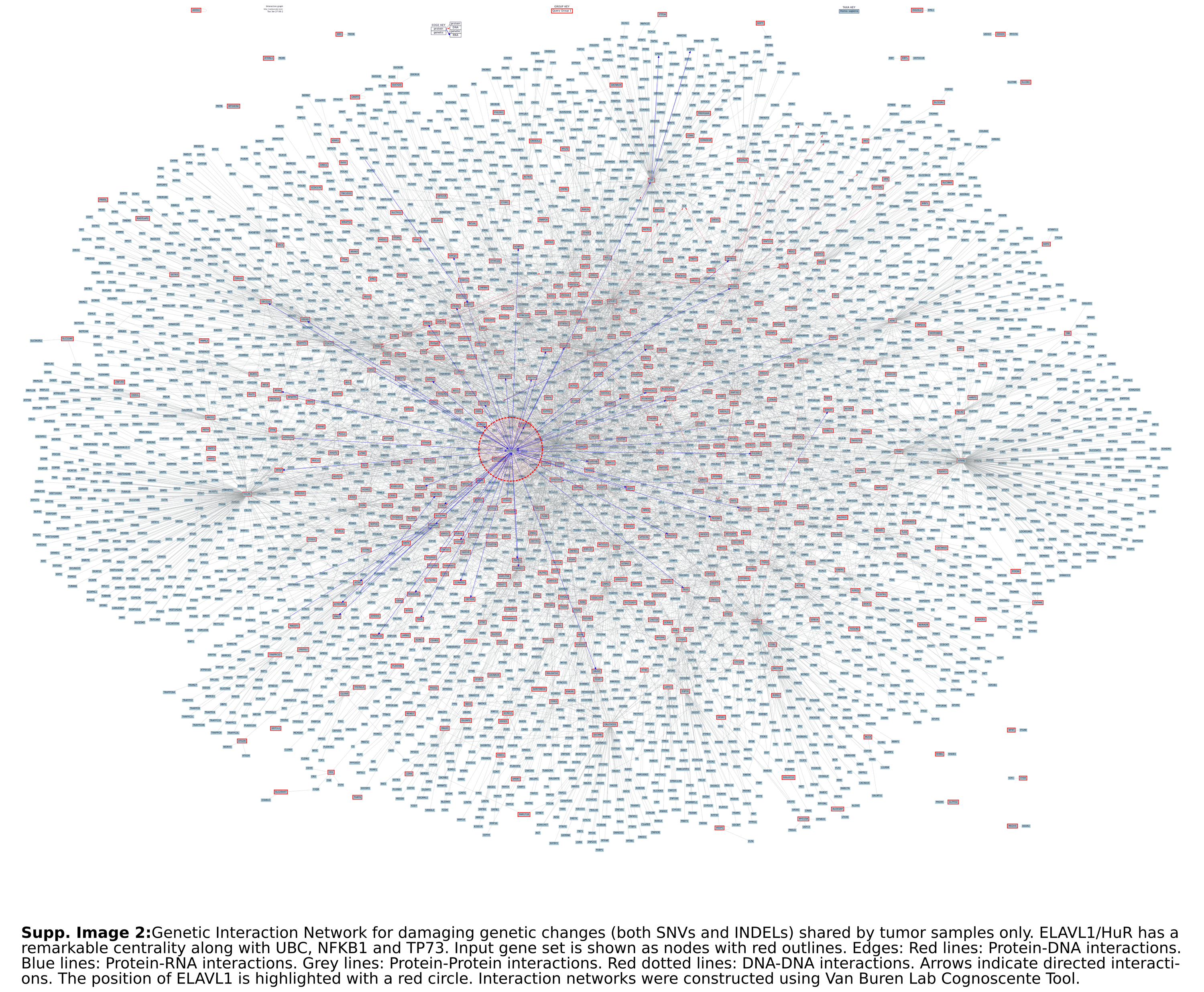

Supplement: Supplementary file 3 [file Image_2.PNG]

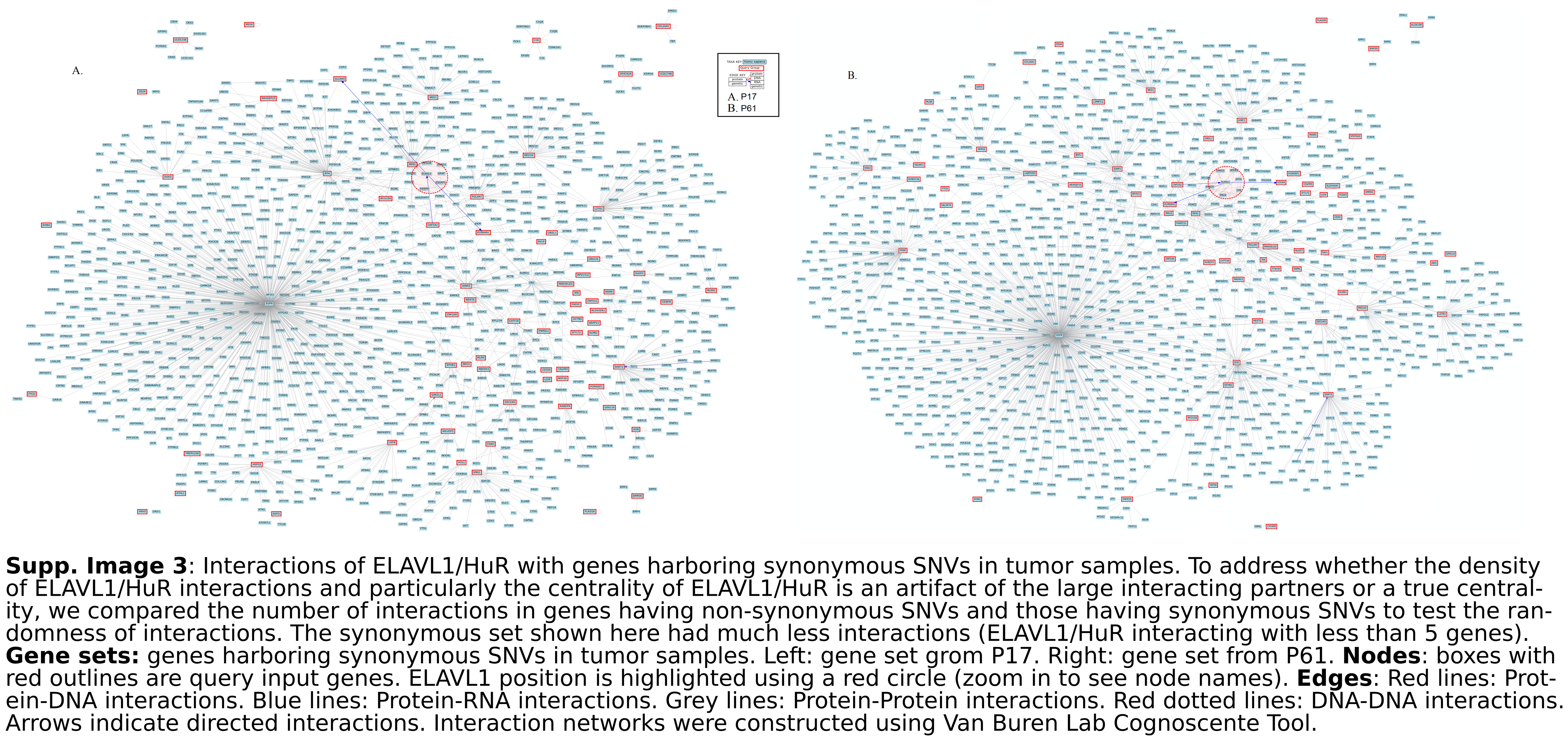

Supplement: Supplementary file 4 [file Image_3.PNG]

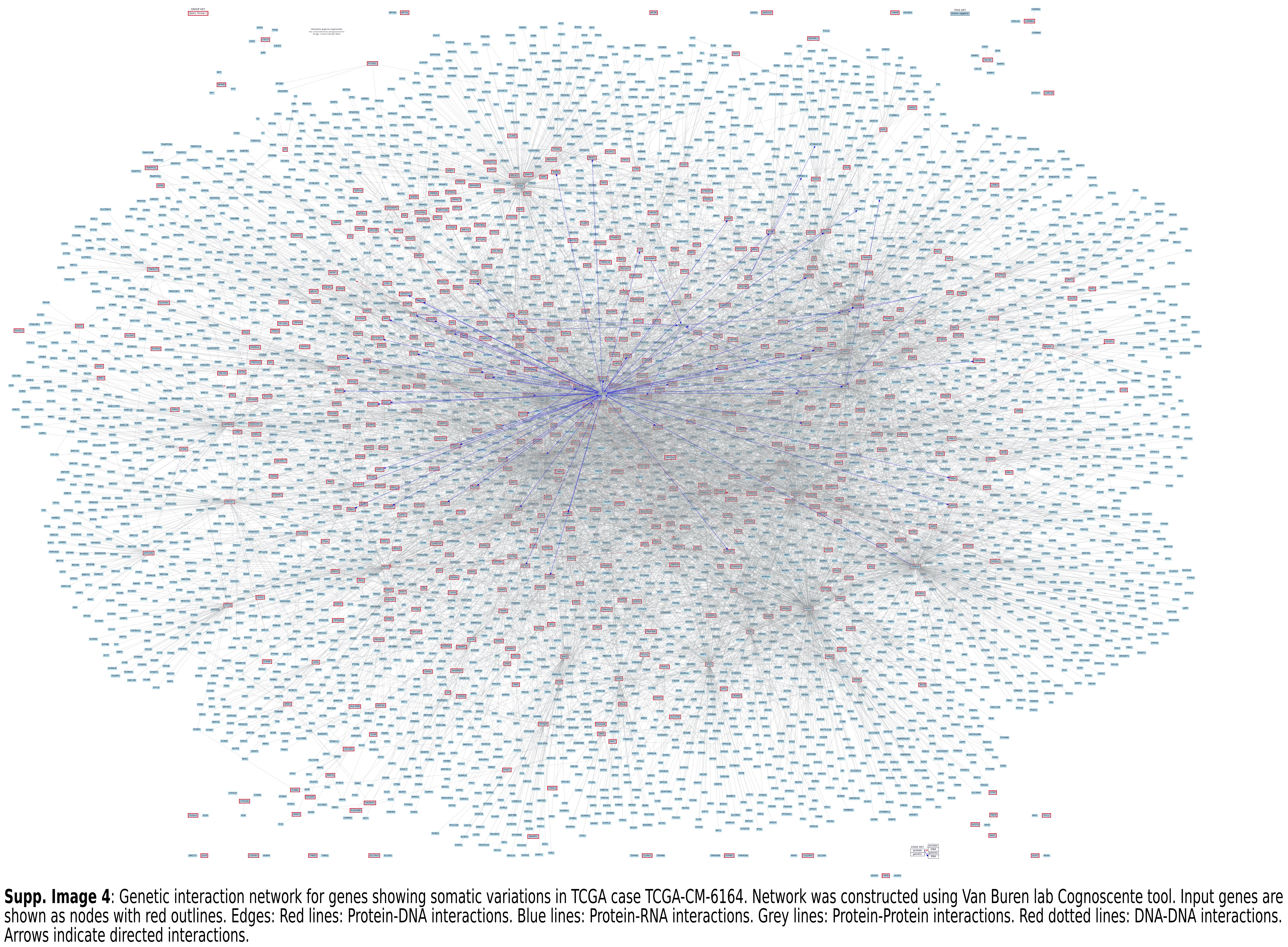

Supplement: Supplementary file 5 [file Image_4.JPEG]

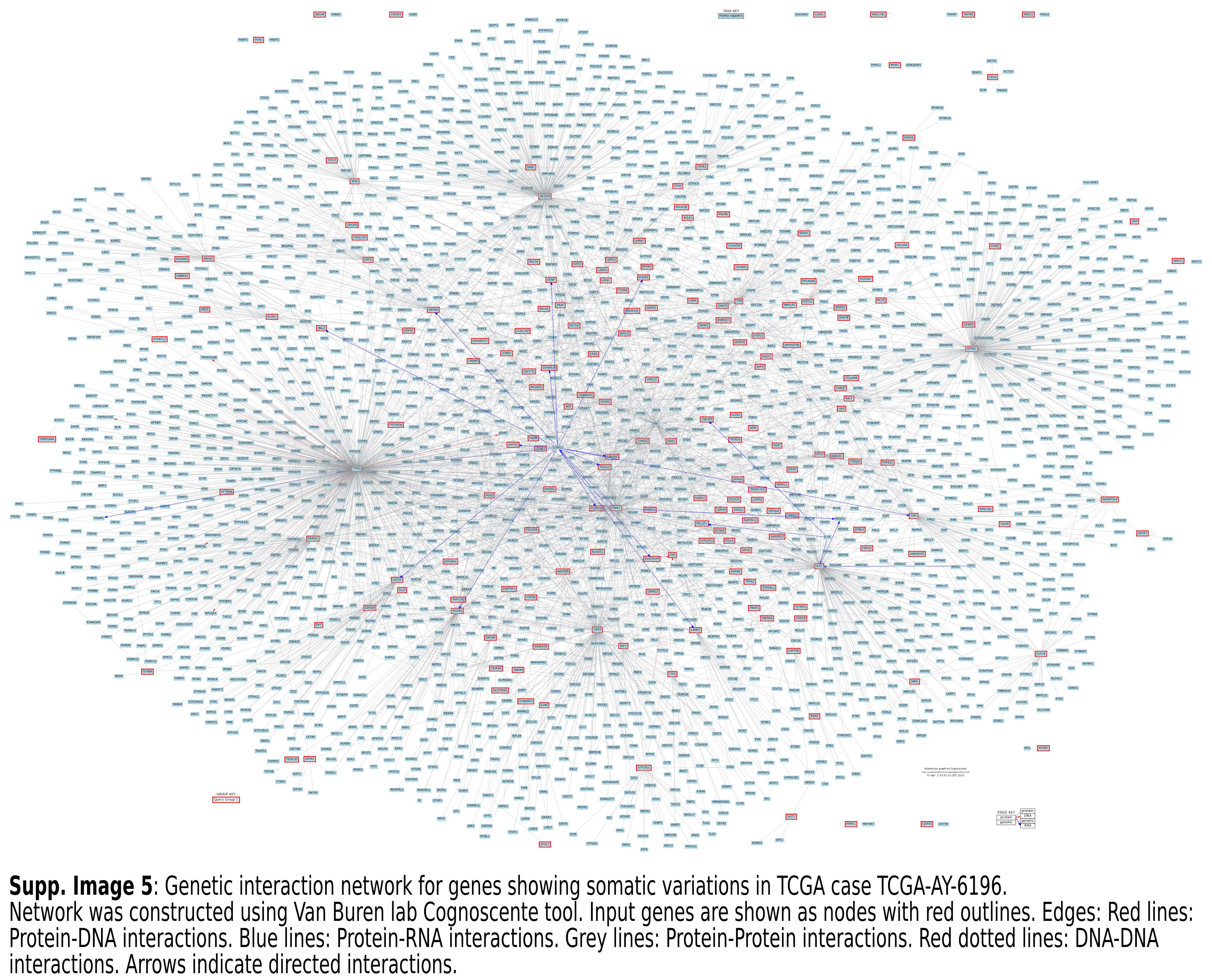

Supplement: Supplementary file 6 [file Image_5.JPEG]

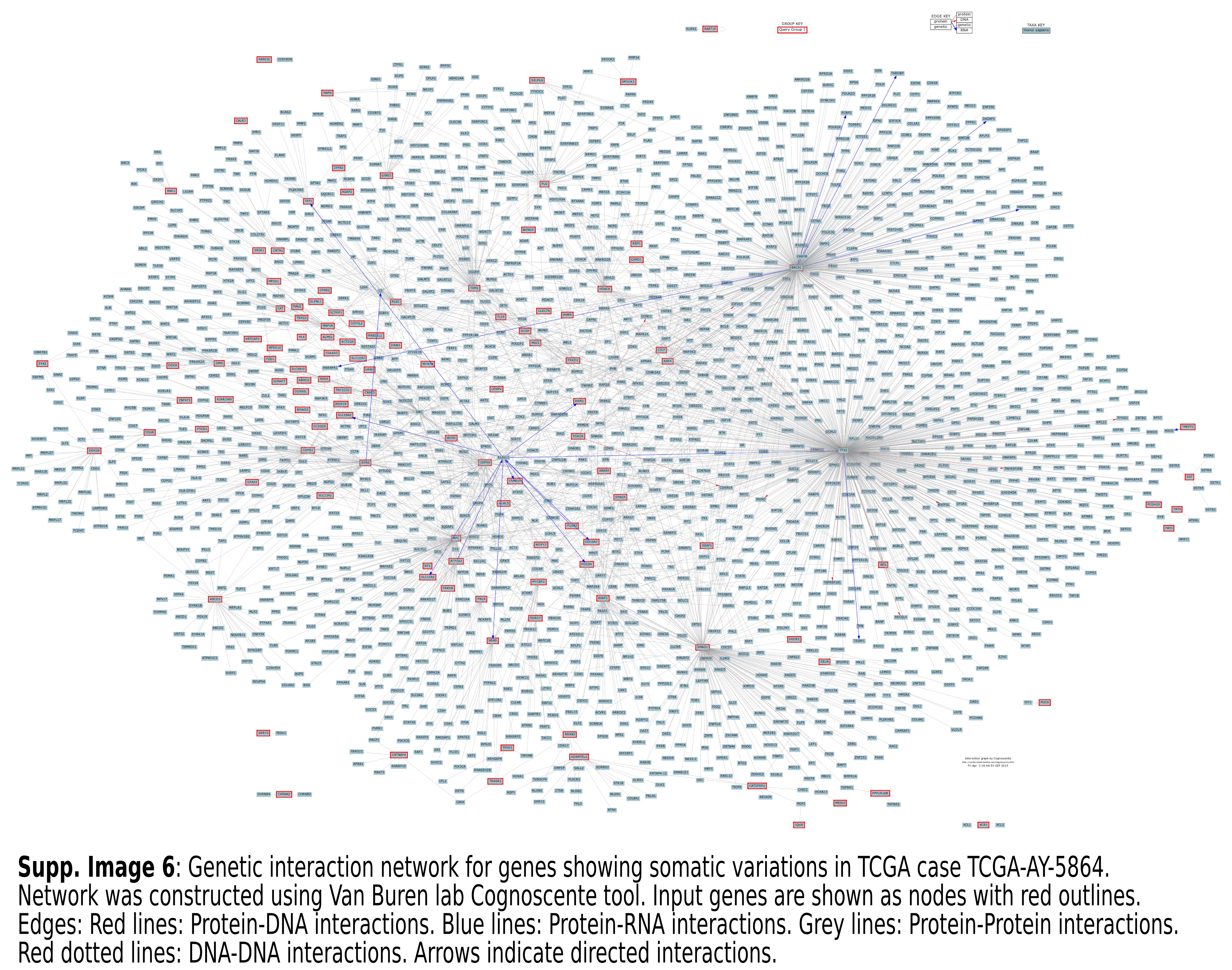

Supplement: Supplementary file 7 [file Image_6.JPEG]

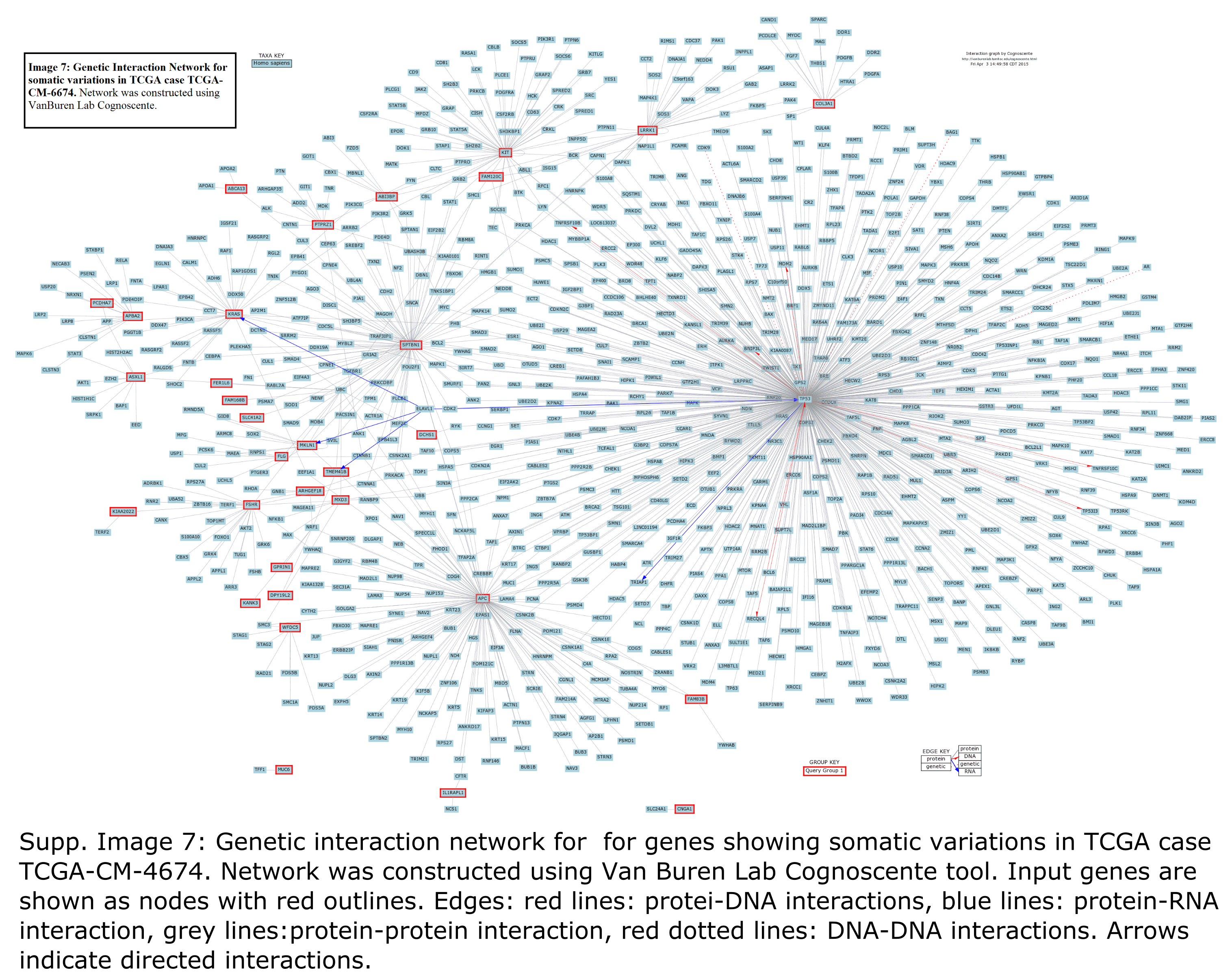

Supplement: Supplementary file 8 [file Image_7.PNG]

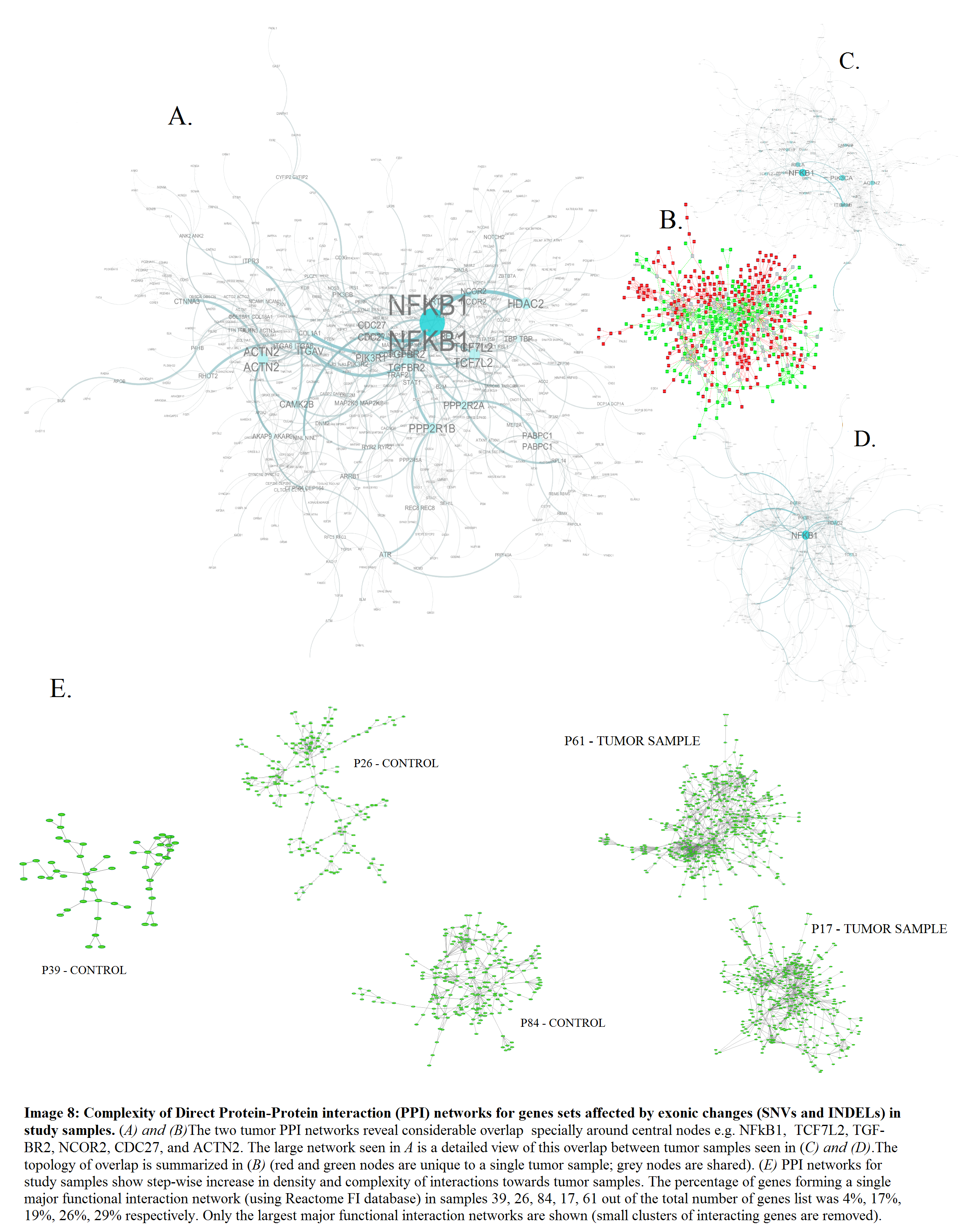

Supplement: Supplementary file 9 [file Image_8.PNG]
